# A rare giant intracranial arachnoid cyst confused the diagnosis and treatment of Wilson disease

**DOI:** 10.1515/tnsci-2022-0213

**Published:** 2022-03-07

**Authors:** Zhang Wenbin, Huang Yeqing, Liu Aiqun, Hong Mingfan, Wei Zhisheng

**Affiliations:** Department of Neurology, School of Clinical Medicine, The First Affiliated Hospital of Guangdong Pharmaceutical University, 19 Nonglinxia Road, Guangzhou, 510080, China

**Keywords:** hepatolenticular degeneration, Wilson disease, intracranial arachnoid cyst

## Abstract

**Background:**

Hepatolenticular degeneration (HLD), also known as Wilson disease (WD), is a rare autosomal-recessive hereditary disease, which is often missed and misdiagnosed because of its various clinical manifestations. And WD is even more rare with giant subarachnoid cysts. In this report, we will provide a case of WD with an intracranial arachnoid cyst (IAC).

**Case description:**

A 27-year-old woman was hospitalized in a traditional Chinese medicine hospital in Guangzhou with the first manifestation of a “slight involuntary tremor of her left upper limb”. There was no improvement after acupuncture treatment, and then she was transferred to another large general hospital in Guangzhou. MRI examination of the head showed “left frontal, parietal and temporal giant subarachnoid cyst” and the patient underwent “left frontotemporal arachnoid cyst celiac shunt operation.” After the operation, the patient’s left limb shaking remained unchanged. Subsequently, the patient was referred to another big hospital in Guangzhou, considered “Parkinson’s disease,” and given “Medopa, Antan” and other treatments. However, the patient’s limb shaking continued to increase and gradually developed to the extremities. At last, the patient was referred to our hospital, combined with the medical history, neurological signs, and auxiliary examination results, improve the examination of corneal K-F ring, blood ceruloplasmin, gene screening, and other tests; the diagnosis was confirmed as hepatolenticular degeneration.

**Conclusion:**

After expelling copper and symptomatic treatment, the condition is improved.

## 1 Introduction

Hepatolenticular degeneration is an autosomal-recessive hereditary disease, early diagnosis and timely treatment are very essential, but because of the diversity and nonspecificity of its early symptoms, it is easy to be misdiagnosed; besides, because of the similarity and overlap of symptoms, it is more likely to be confused when complicated with arachnoid cysts. Therefore, it is often difficult for doctors to identify and deal with patients with WD complicated with IAC in time. In this case report, we will provide a case of WD with a rare giant IAC. From the particularity of this case and the process of diagnosis and treatment, we will probe into the early identification of WD complicated with IAC, the influence factors of aggravation of WD symptoms. and the rationality of treatment, so as to improve the understanding of the disease to clinicians.

### 1.1 Case presentation

The patient, 27 years old, was hospitalized in the First Affiliated Hospital of Guangdong Pharmaceutical University on August 28, 2012, due to “limb shaking for more than one year.” In June 2011, the patient first presented with “slight involuntary shaking of the left upper limb” in a hospital of traditional Chinese medicine in Guangzhou. After acupuncture treatment, there was no improvement, the patient was transferred to another grand hospital in Guangzhou. The MRI of the head showed a “giant arachnoid cyst in the left frontal, parietal and temporal areas.” On August 30, 2011, the patient underwent a “left frontotemporal arachnoid cyst celiac shunt operation.” After the operation, the patient’s left limb shaking remained unchanged. Subsequently, the patient was referred to another big hospital in Guangzhou, misdiagnosed as “Parkinson’s disease,” and given “Medopa, Antan” and other treatments. However, the patient’s limb shaking continued to increase and gradually developed to the extremities. For further diagnosis and treatment, he was admitted to the First Affiliated Hospital of Guangdong Pharmaceutical University (hereinafter referred to as “our hospital”) on August 28, 2012. When transferred to our hospital, the patient was depressed, unstable walking, limb torsion spasm, and inarticulate speech.

The patient has no previous special medical history. Physical examination of the neurological system at the time of transfer was as follows: difficult speech, under the naked eye, no obvious K-F ring in both eyes, shallow nasolabial groove on the right side, left corner of the mouth, right tongue extension, limb muscle strength 4-grade, postural and intentional tremor of both upper limbs, torsion of elbow joint and wrist joint in the right upper limb, ankle hyperextension in both lower extremities, mild atrophy of muscles of extremities, moderate to severe lead tube-like increase in muscle tension of both upper limbs, moderate lead tube increase of muscle tension of both lower limbs, and no obvious abnormality in sensory examination and negative pathological signs were found in bilateral finger nose test and calcaneal tibial test. Meningeal irritation sign was not elicited.

After being transferred to our hospital, MRI showed the following: symmetrical abnormal signal in the thalamus, cerebral peduncle and brain stem, high water pressure signal in T2WI and T2WI, T1WI signal, and giant subarachnoid cyst in the left frontal, parietal and temporal region (size of about 127 mm × 63 mm × 117 mm). Abdominal ultrasound showed the following: combined with clinical consideration, liver cirrhosis caused by hepatolenticular degeneration and enlarged spleen; cholecystitis and cholecystolithiasis have not been excluded. Laboratory examination results were as follows: liver function, renal function, blood routine examination: basically normal, serum copper, 0.21 mg/L (0.7–1.6); and ceruloplasmin, 0.02 g/L (0.16–0.45). Other examinations: under the slit lamp, the K-F ring was positive. The gene detection of hepatolenticular degeneration was c. 2828G > A. Gly943Asp. C. 3443T > C p. Ile1148Thr. SAS 62 score; SDS 75 score.

According to these results, we considered the diagnosis as follows: 1, hepatolenticular degeneration 2, liver cirrhosis 3, left frontotemporal arachnoid cyst abdominal shunt 4, depression. After admission, copper treatment was given: dimercaptopropanesulfonic acid (DMPS): 5 mg/kg was dissolved in 5% glucose solution 500 mL ivgtt qd × 6 days as a course of treatment. Long-term oral administration of penicillamine and zinc gluconate. At the same time, symptomatic drugs were given: medopa, clonazepam, baclofen, venlafaxine, and so on.

**Figure 1:**
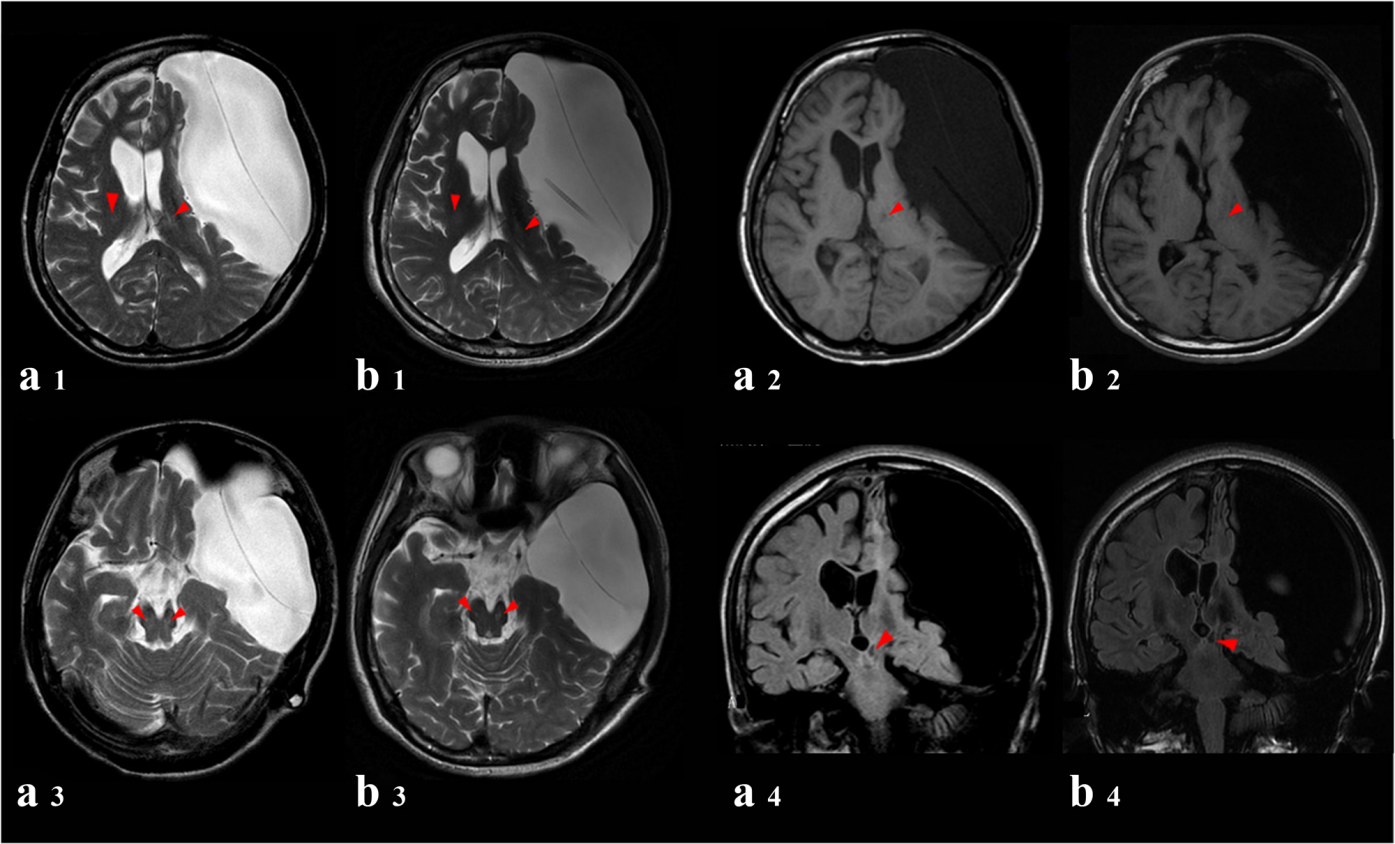
Left arachnoid cyst-after abdominal drainage: the left frontal, parietal and temporal medial plate still showed a 127 mm × 63 mm × 117 mm long T1 long T2 signal shadow, T2WI water pressure low signal, a linear T2WI low signal segmentation shadow, and drainage tube shadow; the boundary between the lesion and the surrounding tissue was clear, the adjacent brain mass was obviously compressed and displaced, the cerebral gyrus and cerebral fissure disappeared, and the flaky edema signal shadow was seen in the compressed brain mass; the adjacent medial plate and plate barrier became thinner and locally enlarged, and the fat signal shadow in the plate barrier disappeared. (a1–a4 and b1–b4). A small patch of the abnormal signal was seen in the left thalamus, with T2WI high signal, T1WI slightly low signal (a2 and b2), T2WI water pressure low signal; symmetrical abnormal signal shadows were seen in bilateral thalamus (a1 and b1), cerebral peduncle (a3 and b3) and brainstem (a4 and b4), T2WI and T2WI water pressure hyperintensity, T1WI and other signals. The midline structure shifted to the right about 7 mm, the bilateral lateral ventricle and the third ventricle were compressed and narrowed, and the residual sulcus was not significantly dilated. No abnormality was found in the rest of the skull.


Follow-up: the patients continued to return to the hospital for reexamination and received systematic DMPS copper expelling therapy every year. The size of arachnoid cyst, compression of adjacent brain matter, and midline shift were similar between 2021-04-28MR (Figure 1(b_1_–b_4_)) and 2014-10-9 MR (Figure 1(a_1_–a_4_)). Symmetrical patchy low signal intensity was found in the bilateral thalamus, caudate nucleus, lentiform nucleus, and cerebral foot, and some of the lesions were reduced. The symptoms such as limb tremor, torsion spasm, and depression were relieved.

**Informed consent:** Informed consent has been obtained from all individuals included in this study.
**Ethical approval:** The research related to human use has been complied with all the relevant national regulations, institutional policies and in accordance with the tenets of the Helsinki Declaration, and has been approved by the authors’ institutional review board or equivalent committee.

## 2 Discussion

Hepatolenticular degeneration (HLD) is a rare single-gene autosomal-recessive hereditary disease with an incidence of only 1 × 100,000–1 × 30,000 [1,2]. Due to the diversity of clinical manifestations and lack of specificity, HLD is often missed and misdiagnosed. According to the related research [3,4,5], about 10% of patients start with mental symptoms. About 40% of patients with WD have initial symptoms and signs of liver cirrhosis. In about 40–50% of patients, neurological symptoms are predominant. Therefore, although the symptoms of WD are multisystem complex, neurological WD patients still account for the majority. The most common neurological symptoms include tremor, dystonia, dysarthria, and parkinsonism.

Tremor is the first clinical manifestation in up to 55% of WD patients with neurologic symptoms [6,7]. This patient starts with only mild limb tremor, which is consistent with the symptomatic characteristics of neurotic WD. However, because the sign of the K-F ring was not obvious at that time, it was misdiagnosed as Parkinson’s disease in other hospitals, which also suggested that the early symptoms of WD were not specific or even asymptomatic, which brought great difficulties to the diagnosis. The characteristic tremor is the wing-beating tremor or flapping tremor. Initially, the tremor also can present as the essential tremor form, in the distal upper extremities, with less severe involvement of the head and legs. Latterly, a progression of tremor amplitude may manifest together with cerebellar or rubral features in all parts of the body [7,8]. In this case, it seems that the patient mainly showed postural tremor rather than typical flapping wing tremor, but it first involved the distal end of one limb and gradually developed to the extremities, which was consistent with the above description.

The most common brain MRI findings of WD patients are symmetrical or asymmetric hyperintensities in T2-weighted images in basal ganglia, thalamus, midbrain, and pontine [9]. The most characteristic MRI sign of WD is the so-called “face of the giant panda” in the midbrain, which occurs in 14–20% of WD patients with neurological manifestations [9,10]. There is no “face of the giant panda” sign in the brain MR of this patient, but the characteristic that symmetrical hyperintensities in T2-weighted images in bilateral thalamus and midbrain also correspond to the diagnosis of WD. Besides, young patients with concurrent dystonia should be highly suspected of WD. Hu et al. [11] also confirmed that when there are young patients with the extrapyramidal system as the main symptoms, the possibility of WD diagnosis should be considered. Considering all these factors, we believe that WD should be the first diagnosis to be considered and perform some relevant check; consequently, the inspection results such as B-ultrasonography of abdomen indicates liver cirrhosis, low ceruloplasmin, positive K-F ring under a slit lamp, and gene detection abnormality also confirm our prediction.

It is noteworthy that the special feature of this case is that there is a huge arachnoid cyst. Because of its space-occupying effect, there may also be symptoms of Parkinson’s syndrome such as limb tremor and tremor [12], which also brings great confusion to the clinical diagnosis. Although the onset of WD and IAC were hidden and the symptoms were similar, we noticed that the postoperative symptoms such as limb torsion, spasm, and unclear speech could not be explained simply by the diagnosis of an arachnoid cyst, and the re-examination of MR after operation showed that the space occupation of IAC was not significantly reduced but the symptoms were significantly improved after expelling copper, which also suggested that the symptoms were related to WD rather than IAC. As far as we are concerned, when we encounter a certain aspect of outstanding performance, we carefully analyze whether the performance is the root cause of clinical symptoms and did not jump to conclusions.

On the other hand, in view of the inverse aggravation of postoperative symptoms, we believe that it is closely related to surgical stress, and a cross-sectional study of traumatic WD patients also suggests that there is a causal relationship between neurological symptoms and trauma, surgery, or emotional events [13]. And WD belongs to a single gene hereditary disease, and the mutation of the ATP7B gene is the main pathogenesis of the ATP7B gene [1,2]. Previous studies have also confirmed that environmental factors play a key role in affecting the penetrance rate of the ATP7B gene [14,15,16], while the environmental factors of the body directly affect the penetrance rate, expression degree, and the disease occurrence speed of abnormal genes. Therefore, we believe that surgical stress accelerates the progression of WD by affecting the penetrance of the ATP7B gene, and we suggest that WD patients should avoid trauma and severe mood swings as far as possible; for WD patients with acute nervous system symptoms, they should receive copper resistance therapy in time after mild trauma, and operation or emotional events in order to obtain a good prognosis.

In view of the surgical problem of WD complicated with giant IAC, Yuqin [17] made a retrospective analysis of 142 children with intracranial large arachnoid cyst who underwent cyst-peritoneal shunt. It was proved that cyst-peritoneal shunt has the characteristics of less surgical trauma, definite clinical effect, and low complication rate. Holst et al. [18] analyzed the curative effects of craniotomy, cystoperitoneal shunt, and endoscopic cystostomy. After 30 months of follow-up, it was found that the recurrence rate of the cyst-celiac shunt was significantly lower than that of the other two methods and the postoperative reaction was mild, so it was considered to be safe and effective. However, it is also reported that the symptoms of giant arachnoid cyst after cyst-celiac shunt did not improve but increased the risk of postoperative complications, and most of the current research subjects are limited to children [19]. The surgical study of giant arachnoid cyst in adults still needs to be confirmed by large sample randomized controlled studies and systematic evidence-based medical evidence. Therefore, we believe that the preoperative evaluation of patients with WD complicated with giant IAC becomes particularly important; first of all, it is necessary to determine whether the patient’s symptoms are related to arachnoid cysts, and if it is not related to IAC, long-term follow-up observation is recommended. If it is related to IAC, we believe that the mode of operation should be carefully chosen under the premise of surgical indications. The symptoms of this patient were aggravated after cyst-shunt, and there was no significant reduction in the IAC volume during long-term follow-up. In the long run, a cyst-peritoneal shunt may not be the best choice, and it still has a high risk of recurrence, blockage, and infection, which will also make the patient face the risk of a second operation. At the same time, surgery also increases the risk of aggravating WD.

## 3 Conclusion

So far, it is very rare for WD to merge with giant IAC. There was no relationship between the two diseases, but the symptoms were misdiagnosed as IAC at the initial stage of the disease, and after a cyst-peritoneal shunt in IAC, the condition of WD was aggravated by stress such as operation and trauma. The thinking brought to us by this case was the following: when we encounter a certain aspect of outstanding performance in the clinic, we need to carefully analyze whether the performance is the root cause of clinical symptoms and do not jump to conclusions.
